# Prevalence and Virulence Characteristics of *Enterococcus faecalis* and *Enterococcus faecium* in Bovine Mastitis Milk Compared to Bovine Normal Raw Milk in South Korea

**DOI:** 10.3390/ani12111407

**Published:** 2022-05-30

**Authors:** Hyeon-Jin Kim, Hye-Young Youn, Hye-Jeong Kang, Jin-San Moon, Yong-Seok Jang, Kwang-Young Song, Kun-Ho Seo

**Affiliations:** 1 Center for One Health, College of Veterinary Medicine, Konkuk University, 120 Neungdong-ro, Gwangjin-gu, Seoul 05029, Korea; khj970923@naver.com (H.-J.K.); younv_v123@naver.com (H.-Y.Y.); ryej9@hanmail.net (Y.-S.J.); drkysong@gmail.com (K.-Y.S.); 2 Bacterial Disease Division, Animal and Plant Quarantine Agency, 177 Hyeksin 8-ro, Gimcheon-si 39660, Gyeonsangbuk-do, Korea; saeil3@daum.net (H.-J.K.); moonjs727@korea.kr (J.-S.M.)

**Keywords:** *Enterococcus* spp., bovine mastitis milk, bovine normal raw milk, virulence factors, antimicrobial resistance

## Abstract

**Simple Summary:**

Characterization of *Enterococcus* spp. among bovine mastitis pathogens is important in terms of the dairy industry and public health domain. The present study aimed to characterize virulence properties such as virulence genes (*esp*, *asa1*, *gelE*, and *cylA*), biofilm, gelatinase, hemolysis, and antimicrobial resistance and to compare them between bovine mastitis milk and bovine normal raw milk. Our results demonstrated that *Enterococcus faecalis* and *Enterococcus faecium* isolated from bovine mastitis milk exhibited higher virulence properties than those isolated from bovine normal raw milk. *E. faecalis* (90 isolates) was present at a significantly higher rate than *E. faecium* (32 isolates) and was more abundant in virulence genes. Furthermore, after analyzing the correlation between the virulence genes and the corresponding phenotype, we found that *gelE* and *esp* were involved in strong biofilm formation and the *gelE* was involved in gelatinase production. Taken together, *E. faecalis* in bovine mastitis milk should be monitored to control bovine mastitis and improve public health.

**Abstract:**

*Enterococcus* spp. are pathogens that cause environmental mastitis and are difficult to eliminate owing to their resistance to antibiotics. To compare the virulence characteristics of isolates from bovine mastitis milk (BMM) and bovine normal raw milk (NRM), we isolated *Enterococcus* spp. from 39 dairy farms in South Korea from 2015–2020. A total of 122 *Enterococcus* spp. were identified, with *Enterococcus faecalis* (73.8%) accounting for the majority, followed by *Enterococcus faecium* (26.2%). *E. faecalis* isolated from BMM harbored *gelE*, *asa1*, *esp*, and *cylA* genes with a prevalence of 85.7, 71.4, 54.3, and 30.0%, respectively. These genes were significantly more abundant in BMM than in NRM, except for *asa1* (*p* < 0.0001). Interestingly, strong biofilm and gelatinase formation was predominately observed for BMM isolates and this was significantly correlated to the presence of *esp* and *gelE* genes (*p* < 0.05). BMM isolates demonstrated higher resistance to tetracycline (59.3%), followed by chloramphenicol (21.0%), rifampicin (18.5%), doxycycline (4.9%), ciprofloxacin (1.2%), and nitrofurantoin (1.2%), than those from NRM. *E. faecalis* harboring *esp*, *gelE*, and *cylA* may be causative agents for bovine mastitis and act as a reservoir for the transmission of virulence factors to humans.

## 1. Introduction

Bovine mastitis is a disease that affects dairy animals, resulting in a decrease in both milk quality and production [1]. Bovine mastitis pathogens are classified as either “contagious” or “environmental” with *Staphylococcus aureus*, *Streptococcus agalactiae*, and *Mycoplasma* spp. being representative pathogens of contagious mastitis [2]. In contrast, enterococci are environmental causative agents of mastitis, with infections caused primarily by *Enterococcus faecalis* (approximately 80%) and *Enterococcus faecium* (approximately 10–15%) [3,4].

*Enterococcus* spp. are Gram-positive bacteria that live in the gastrointestinal tracts of humans and other animals. In recent decades, they have emerged as major causes of nosocomial infections [5]. Due to their ability to adapt to adverse conditions, *Enterococcus* spp. can survive in the environment for long periods of time and can infect the mammary glands [3]. Although ampicillin (AMP), gentamicin, penicillin, tetracycline (TET), and tylosin (TYL) are antimicrobial agents approved and previously used for the treatment of bovine mastitis in South Korea, their therapeutic success is limited by antimicrobial resistance [6]. Furthermore, *Enterococcus* spp. serve as a reservoir of antimicrobial resistance genes, allowing them to be transmitted to humans via the food chain [7]. Our previous studies identified *E. faecalis* and *E. faecium* resistant to TET, erythromycin (ERY), ciprofloxacin (CIP), high-level streptomycin, levofloxacin, and linezolid in ripened cheese products [8]. The antibiotic-resistant *Enterococcus* spp. can cause various infections in humans, including urinary tract infection, endocarditis, meningitis, and bacteremia, all of which can be difficult to treat with antibiotics [9,10].

The ability of *Enterococcus* spp. to form biofilms is considered a virulence factor that contributes to its antibiotic resistance [11]. A mature biofilm can endure 10 to 1000 times greater concentrations of antimicrobial agents than those required to kill planktonic bacteria [12]. The genes involved in biofilm formation include *esp* (enterococcal surface protein-encoding gene), *asa1* (aggregation substance-encoding gene), and *gelE* (gelatinase-encoding gene) [13]. Gelatinase can hydrolyze gelatin, hemoglobin, collagen, casein, and other bioactive compounds [14]. Additionally, cytolysin (encoded by *cylA)* can induce the hemolysis of human red blood cells [15].

Because virulence genes can be carried on mobile genetic elements, *Enterococcus* spp. in milk can contribute to the spread of these genes in humans [16]. There is a lack of information regarding virulence and antimicrobial resistance in isolates of bovine mastitis milk (BMM) compared to those in bovine normal raw milk (NRM). To the best of our knowledge, this is the first study that aimed to characterize and compare the virulence properties of *Enterococcus* spp. isolated from BMM and NRM. Therefore, the purpose of this study was to determine the virulence characteristics of isolates of *Enterococcus* spp. from BMM and NRM by comparing virulence factors (genes and biofilm, gelatinase, and hemolysin formation), correlation between virulence phenotype and genotype, and antimicrobial resistance.

## 2. Materials and Methods

### 2.1. Sample Collection and Isolation of E. faecalis and E. faecium

*E. faecalis* and *E. faecium* were isolated by collecting at least one BMM each from 36 dairy farms in South Korea between 2015 and 2020. For comparison of virulence properties with BMM, *E. faecalis* and *E. faecium* were isolated from at least one NRM from each of three dairy farms in South Korea in 2020. The somatic cell count (SCC) values of the BMM and NRM were measured using the Fossmatic System 4000 (Foss Electric, Hillerød, Denmark). Milk with SCC ≥ 200,000 cells/mL was considered positive for bovine mastitis and was prohibited for human consumption, while that with SCC < 200,000 cells/mL was considered normal. To isolate *E. faecalis* and *E. faecium* from BMM and NRM, milk samples were inoculated on blood agar (Komed, Gyeonggi, South Korea) and incubated at 37 °C for 24 h. After incubation, round white suspected colonies were identified by Matrix-Assisted Laser Desorption Ionization–Time of Flight Mass Spectrometer (MALDI-TOF MS; bioMérieux, Marcy l’Etoile, France), according to the manufacturer’s instructions. The isolates were stored at −80 °C in tryptic soy broth (Sigma-Aldrich, St. Louis, MO, USA) with 25% sterile glycerol for further analysis.

### 2.2. DNA Extraction and Screening for Virulence Genes

All *Enterococcus* spp. were cultivated in brain heart infusion (BHI) broth (Sigma-Aldrich, St. Louis, MO, USA) at 37 °C. DNA extraction was performed using a QIAamp DNA Mini Kit (Qiagen, Hilden, Germany), following the manufacturer’s instructions. The extracted DNA was screened for the presence of *esp*, *asa1*, *gelE*, and *cylA* genes using conventional polymerase chain reaction (PCR) with the corresponding primers (Supplementary Table S1) [17]. Primer sets were synthesized and purchased from Bionics (Seoul, Korea). Each PCR amplification was performed in a 20 µL reaction volume, containing 1 µL of template DNA, 2 µL of total primer, and 17 µL of distilled water using the Maxime PCR PreMix Kit (*i*-Taq) (iNtRON Biotechnology, Seongnam, South Korea). An initial activation step at 95 °C for 15 min was followed by 30 cycles of denaturation (94 °C, 1 min), annealing (temperatures indicated in Supplementary Table S1, 1 min), and extension (72 °C, 1 min), followed by one cycle of 10 min at 72 °C [17]. *E. faecalis* KCTC 3206 (ATCC 19433) and KCTC 3511 (ATCC 29212) obtained from the Korean Collection for Type Cultures (KCTC; Jeongeup, South Korea) were used as positive controls.

### 2.3. Phenotype of Virulence Characteristics

#### 2.3.1. Biofilm Formation

The assessment of biofilm formation was based on a method described by Stepanović et al. (2007) with some modifications [18]. Briefly, bacteria were cultured at 37 °C in BHI agar (Sigma-Aldrich, St. Louis, MO, USA) for 24 h. Each culture was then suspended in BHI broth (Sigma-Aldrich, St. Louis, MO, USA) supplemented with 2% glucose (Sigma-Aldrich, St. Louis, MO, USA) to prepare a 0.5 McFarland standard suspension (about 10^7^ colony forming units (CFU)/mL). Each well of a sterile 96-well culture plate (SPL Life Sciences, Pocheon-si, Gyeonggi-do, Korea) was filled with a 200 µL bacterial suspension. Subsequently, the plate was incubated for 24 h at 37 °C and the broth was carefully removed. The wells were gently washed three times with sterile phosphate buffered saline (PBS; Sigma-Aldrich, St. Louis, MO, USA). Following every washing step, the adherent biofilm layer formed in each well was stained with 200 µL 0.1% crystal violet (Sigma-Aldrich, St. Louis, MO, USA) solution in distilled water for 20 min at 25 °C. After each staining step, the well was washed with PBS (Sigma-Aldrich, St. Louis, MO, USA) until the washing was free of stain, and the optical density (OD) was measured using a Multiskan FC (Thermo Fisher Scientific, Shanghai, China) at 595 nm. The cut-off OD (ODc) was defined by the negative control. Based on the bacterial biofilm-forming activity, the isolates were classified as follows: OD ≤ ODc = non-biofilm formation, ODc < OD ≤ 2 ODc = weak biofilm formation, 2 ODc < OD ≤ 4 ODc = moderate biofilm formation, and OD > 4 ODc = strong biofilm formation.

#### 2.3.2. Gelatinase Production

For the detection of gelatinase activity, *Enterococcus* spp. were inoculated on nutrient gelatin (KisanBio Co., Ltd., Seoul, Korea). *S. aureus* ATCC 25923 was used as the positive control for gelatin hydrolysis experiments, while *Escherichia coli* KCTC 2571 was used as the negative control in the gelatin hydrolysis experiment. Pure cultures were individually stabbed into tubes, incubated at 37 °C for 48 h, and then held at 4 °C for 30 min. In tubes where an organism produced sufficient gelatinase, the gelatin remained liquefied upon cooling.

#### 2.3.3. Hemolysin Production

Hemolysis activity was determined on Columbia agar with 5% sheep blood (bioMérieux, Marcy l’Etoile, France) and incubated at 37 °C for 48 h. The presence or absence of clearing zones around the colonies was interpreted as β-hemolysis (positive hemolysis) or γ-hemolysis (negative hemolysis), respectively. *S. aureus* ATCC 25923 was used as the positive control.

### 2.4. Antimicrobial Resistance

Isolates were investigated for their susceptibility towards nine different antimicrobials using the disc diffusion method in accordance with Clinical and Laboratory Standards Institute (CLSI) guidelines [19]. Briefly, a 0.5 McFarland standard suspension was prepared for each isolate and the isolate was cultured uniformly on Muller-Hinton agar (Oxoid, Basingstoke, UK) by streaking the swab in a back-and-forth motion. Antimicrobial discs (Oxoid, Basingstoke, UK) individually loaded with AMP (10 μg), chloramphenicol (30 μg; C), CIP (5 μg), doxycycline (30 μg; DOX), ERY (15 μg), nitrofurantoin (300 μg; N), rifampicin (5 μg; RIF), TET (30 μg), and vancomycin (30 μg; VAN) were placed on the agar plates, followed by incubation for 24 h at 37 °C. The inhibition zone size was measured using a ruler. Susceptibility or resistance of the organism to each tested drug was determined using *S. aureus* ATCC 25923 as the control strain [19]. Multidrug resistance (MDR) was defined as acquired resistance to at least one agent in three or more antimicrobial classes.

### 2.5. Statistical Analysis

All data were analyzed using Pearson’s chi-square test. GraphPad Prism version 8.0 (GraphPad Software Inc., La Jolla, CA, USA) was used for data analysis; a *p*-value < 0.05 was considered significant.

## 3. Results

### 3.1. Identification of E. faecalis and E. faecium from BMM and NRM

**Figure 1:**
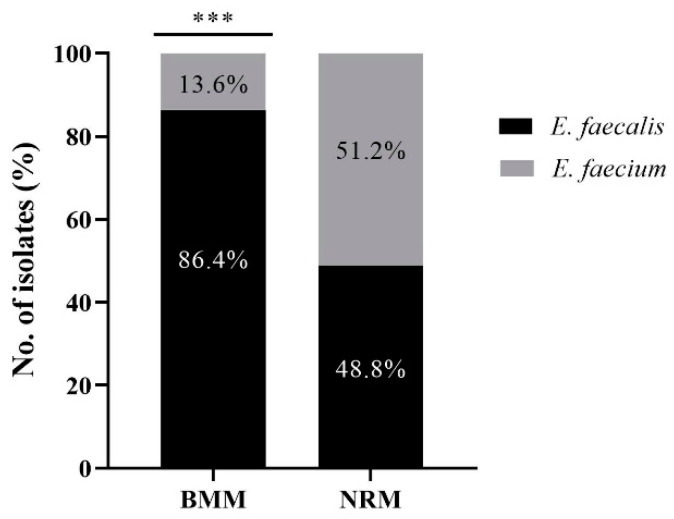
Comparison of the prevalence of *Enterococcus faecalis* and *Enterococcus faecium* isolates in bovine mastitis milk (BMM; *n* = 81) and bovine normal raw milk (NRM; *n* = 41). *** indicates significant difference in the prevalence rate of *E. faecalis* and *E. faecium* isolates in BMM (*p* < 0.0001; Pearson’s chi-square test). BMM: bovine mastitis milk, NRM: bovine normal raw milk.

In total, 81 and 41 *Enterococcus* spp. were isolated from BMM and NRM, respectively (Figure 1). According to the MALDI-TOF MS results, *E. faecalis* (70/81, 86.4%) was identified in BMM at an approximately six-fold higher rate than *E. faecium* (11/81, 13.6%) (*p* < 0.0001). In NRM, 21 isolates were identified as *E. faecium* (21/41, 51.2%), while the remaining isolates were identified as *E. faecalis* (20/41, 48.8%).

### 3.2. Detection of Virulence Genes

Among the 70 *E. faecalis* isolated from BMM samples, 85.7, 71.4, 54.3, and 30.0% harbored the *gelE*, *asa1*, *esp*, and *cylA* genes, respectively (Table 1). All 70 of the isolates identified as *E. faecalis* harbored at least one virulence gene and 20 of these isolates harbored all four virulence genes. Conversely, none of the virulence genes were detected in 10 of the 11 isolates identified as *E. faecium* in BMM, while only one isolate harbored both *asa1* and *gelE*. Furthermore, in the case of NRM, none of the *E. faecium* harbored any of the virulence genes, whereas *E. faecalis* harbored *asa1* (16/20, 80.0%), *gelE* (12/20, 60.0%), and *esp* (5/20, 25.0%) (Table 1). Notably, *esp*, *gelE*, and *cylA* were significantly more abundant in *E. faecalis* isolated from BMM than in those from NRM (*p* < 0.0001).

### 3.3. Biofilm, Gelatinase, and Hemolysin Production

**Figure 2:**
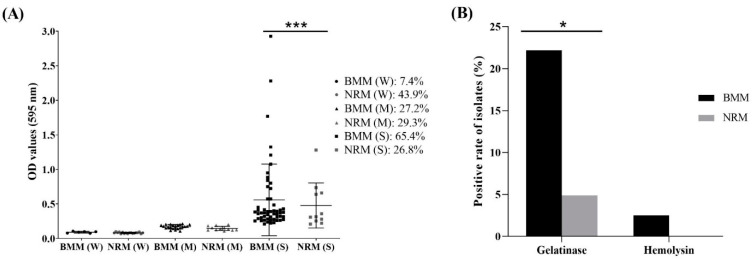
Distribution of *Enterococcus faecalis* and *Enterococcus faecium* isolated from bovine mastitis milk (BMM) and bovine normal raw milk (NRM) according to their virulence factors. (**A**) Biofilm formation ability (OD values) and (**B**) gelatinase and hemolysin production. Biofilm formation criteria comprised non-biofilm formation (optical density (OD) ≤ 0.052), weak biofilm formation (0.052 < OD ≤ 0.104), moderate biofilm formation (0.104 < OD ≤ 0.208), and strong biofilm formation (OD > 0.208). BMM: bovine mastitis milk, NRM: bovine normal raw milk, W: weak, M: moderate, S: strong. *** indicates significant difference in strong biofilm formation between the BMM (S) and the NRM (S) group (*p* < 0.0001; Pearson’s chi-square test). * indicates significant difference in gelatinase production between the BMM and the NRM groups (*p* < 0.05; Pearson’s chi-square test).

The results of biofilm formation of the 122 *Enterococcus* spp. are shown in Figure 2A. Biofilm formation criteria were determined as follows: OD ≤ 0.052 = non-biofilm formation, 0.052 < OD ≤ 0.104 = weak biofilm formation, 0.104 < OD ≤ 0.208 = moderate biofilm formation, and OD > 0.208 = strong biofilm formation. All isolates formed a biofilm, with biofilm OD values ranging from 0.076 to 2.927 for BMM and 0.067 to 1.280 for NRM. Among the isolates from BMM, 53/81 (65.4%), 22/81 (27.2%), and 6/81 (7.4%) isolates formed strong, moderate, and weak biofilm, respectively. Among those isolated from NRM, 18/41 (43.9%), 12/41 (29.3%), and 11/41 (26.8%) isolates formed weak, moderate, and strong biofilms, respectively. The isolates from BMM demonstrated a significantly higher rate of strong biofilm formation than isolates from NRM (*p* < 0.0001).

Furthermore, according to Figure 2B, the isolates from BMM showed significantly higher positivity rates for gelatinase production than isolates from NRM, with 18/81 (22.2%) and 2/41 (4.9%), respectively (*p* < 0.05). In the case of hemolysin production, 2/81 (2.5%) of isolates from BMM were observed, whereas all isolates from NRM were negative.

### 3.4. Correlation between Virulence Genes and Corresponding Phenotype

The results of the phenotype of biofilm formation, gelatinase, and hemolysin *in vitro* and detection of related virulence genes by conventional PCR are presented in Table 2. Simultaneous virulence gene expression and strong biofilm formation were observed in isolates from BMM harboring *gelE* (43/81, 53.1%), *asa1* (37/81, 45.7%), and *esp* (35/81, 43.2%), whereas simultaneous non-expression was observed in the isolates harboring *esp* (25/81, 30.9%), *asa1* (14/81, 17.3%), and *gelE* (10/81, 12.3%). However, in the case of isolates from NRM, simultaneous expression of virulence genes and strong biofilm formation were observed in isolates harboring *gelE* (6/41, 14.6%), *asa1* (5/41, 12.2%), and *esp* (5/41, 12.2%), whereas simultaneous non-expression was observed by the isolates with *esp* (30/41, 73.2%), *gelE* (24/41, 58.5%), and *asa1* (19/41, 46.3%). Furthermore, the *esp* and *gelE* genes were significantly related to strong biofilm formation (*p* < 0.05).

Comparing the detection results of gelatinase and *gelE* gene of isolates from BMM, simultaneous expression was observed in 18 isolates (18/81, 22.2%), whereas simultaneous non-expression was observed in 20 isolates (20/81, 24.7%). In the case of isolates from NRM, simultaneous expression was demonstrated in 2 isolates (2/41, 4.9%), whereas simultaneous non-expression was demonstrated in 20 isolates (20/41, 48.8%). In addition, in the case of isolates from BMM, the *cylA* gene was positive for 21 isolates, with only 2 isolates showing β-hemolysis, whereas all isolates from NRM were negative for both of these. In the present study, all isolates from BMM and NRM positive for gelatinase and hemolysin activity were *E. faecalis*, and in the case of phenotypic expression, related genes (*gelE* and *cylA*) were also detected. Gelatinase production was significantly associated with the *gelE* gene, whereas hemolysin was not associated with the *cylA* gene (*p* < 0.05).

### 3.5. Antimicrobial Resistance of E. faecalis and E. faecium

The results of the antimicrobial resistance analysis using the disc diffusion method for *E. faecalis* and *E. faecium* isolates from BMM and NRM are shown in Table 3. Among the 81 isolates from BMM, the highest resistance rate was shown against TET (48/81, 59.3%), followed by ERY (22/81, 27.2%), C (17/81, 21.0%), RIF (15/81, 18.5%), DOX (4/81, 4.9%), CIP (1/81, 1.2%), and N (1/81, 1.2%). Among MDR isolates from BMM, the antimicrobial resistance patterns of *E. faecalis* were C-ERY-TET (5/13, 38.5%), C-DOX-ERY-TET (3/13, 23.1%), C-RIF-TET (2/13, 15.4%), C-ERY-RIF-TET (1/13, 7.7%), and ERY-RIF-TET (1/13, 7.7%). Interestingly, one *E. faecium* isolate from BMM demonstrated a DOX-ERY-RIF-TET (1/13, 7.7%) antimicrobial resistance pattern. However, *E. faecalis* and *E. faecium* isolates from NRM showed 14/41 (34.1%), 7/41 (17.1%), and 6/41 (14.6%) resistance to ERY, TET, and RIF, respectively. Interestingly, two MDR isolates from NRM showed strong biofilm-forming ability (OD > 0.208), and the antimicrobial resistance pattern was ERY-RIF-TET for the two isolates.

## 4. Discussion

A high SCC value (≥200,000 cells/mL) indicates an increase in leukocytes, reflecting an inflammatory reaction to mastitis, and is used as a general indicator of milk safety [20]. In the present study, *E. faecalis* and *E. faecium* were isolated from BMM with SCC values of 5–6 log cells/mL. Milk with an SCC value > 5 × 10^5^ cells/mL is unsuitable for human consumption in South Korea [20]. Although the intake of BMM is prohibited, *Enterococcus* spp. survive in herd bedding and housing, allowing transmission to humans through cross-contamination in milk bulk tanks, cheese vats, curd cutters, and to workers during milking [10,21,22]. Consistent with our study, the majority of *Enterococcus* spp. isolated from BMM and milk bulk tanks were identified as 91.13% and 90.24% *E. faecalis*, respectively [3,16]. Therefore, it might be effective and economical to intensively manage *E. faecalis* to reduce the damage caused by *Enterococcus* spp. to the dairy industry.

Since virulence genes of *Enterococcus* spp. can be transferred via plasmids, there is a risk that normal strains lacking genes for virulence factors may acquire these genes through conjugation [11]. Furthermore, the virulence factors of enterococci can enhance enterococcal infections and contribute to potential pathogenesis and disease severity in humans and other animals [11]. The *gelE*, which was the most abundant virulence gene in the current study, is involved in gelatinase production and can play an important role in the invasion and dissemination of *Enterococcus* spp. across the intestinal cell layer [14]. Furthermore, *gelE* has appeared more frequently in clinical isolates than in non-infectious strains [23]. Hemolysin (commonly called cytolysin and encoded by *cylA*) is an important factor in determining the lethality of endocarditis and both cytolysin and aggregation substance (encoded by *asa1*) was associated with lethality in 55% of animals when expressed [15]. In a previous study, *gelE*, *esp*, and *cylA* were reportedly present in 77.2, 68.4, and 54.5% of clinical *Enterococcus* spp. isolates, respectively [24]. In the present study, all virulence genes were more abundant in *E. faecalis* than in *E. faecium*; among these genes, *gelE*, *esp*, and *cylA* genes were significantly more abundant in BMM isolates than in NRM isolates, supporting the claim that virulence genes enhance enterococcal infections.

The genotype-phenotype discrepancies were observed for some isolates, and despite genetic evidence, a lack of phenotypic expression indicates the presence of silent genes [25]. Various environmental factors, such as ion concentration, temperature, and osmolarity of the medium, can downregulate genetic expression and this may have a negative effect on gene production [25]. However, silent genes may be activated by the balance of organisms in the intestinal flora, conditions in the gastrointestinal tract, and effects of bacterial synergism, as well as the presence and persistence of large numbers of viable enterococci *in vivo* [11]. The expression of *cylA* is greatly affected by the culture conditions and the hemolytic reaction was more common in clinical isolates than in food isolates [11,26]. Based on our findings, even if the phenotype is not expressed *in vitro*, it may occur in animals or humans, reinforcing the need to monitor the virulence genes of *E. faecalis* in BMM.

Strong biofilms protect microorganisms from extreme pH, osmolality, nutrient deficiencies, mechanical, and shear forces, as well as antimicrobial agents and host immune cells [27]. Similar to the results of Medeiros et al. (2014) the rate of strong biofilm formation of *E. faecalis* and *E. faecium* analyzed in the present study was significantly higher in isolates from BMM than in isolates from NRM [24]. Furthermore, many recent studies have investigated whether virulence genes affect biofilm formation [13,28,29,30]. The *esp* and *gelE* showed a significant association with strong biofilm formation in our study; in previous investigations, knockout mutants of these genes formed weak biofilm compared to the wild-type strains [13,31]. The gelatinase (encoded by *gelE*) modifies bacterial cell surface hydrophobicity to enhance biofilm formation through its ability to cleave substrates at hydrophobic residues [32]. Our study demonstrated that the presence of *esp* and *gelE* in isolates from BMM and NRM was significantly associated with strong biofilm formation.

Resistance to TET and ERY has reportedly increased in South Korea isolates from livestock in the last few decades, attributable to the use of these antimicrobial agents to treat clinical and subclinical mastitis [33,34]. Interestingly, C-resistant *Enterococcus* spp. generally show co-resistance to ERY, likely due to the adjacent location of ERY and C resistance genes in the same plasmid [35]. Although ERY is less marketed to the dairy industry than other antibiotics, ERY resistance likely stems from resistance to other members of the macrolides class, such as TYL, which is used to treat mastitis in South Korea [6,16]. Różańska et al. (2019) showed that among 426 *Enterococcus* spp. isolates, those resistant to ERY, C, and TYL showed similar resistance rates at 208 (48.83%), 191 (44.84%), and 181 (42.49%), respectively [3]. RIF is usually used in the treatment of MDR infections in combination with other antimicrobial agents to achieve a synergistic effect, which can limit treatment options for MDR pathogens [36]. Consistent with these facts, our results indicated high resistance rates to TET, ERY, C, and RIF, and 7 out of 15 MDR isolates included RIF resistance. The use of antibiotics in dairy cows presumably resulted in the detection of significantly more antibiotic-resistant *Enterococcus* spp. in BMM, as the predominance of antibiotic-resistant *Enterococcus* spp. results in bovine mastitis.

Considering its presence in the dairy industry, the serious human infection, and its virulence factors that can spread through cross-contamination, the findings of the current study support the importance of vigilant monitoring of *E. faecalis* in dairy products. Further studies using pulsed field gel electrophoresis or other subtyping tools, such as whole genome sequencing, are needed to confirm that the virulence genes and antibiotic resistance of isolates from raw milk are consistent with those found in farm environments and dairy products.

## 5. Conclusions

Our results suggest that isolates from BMM have higher positivity rates for virulence genes, exhibit stronger biofilm, gelatinase, and hemolysin formation, and have higher rates of antimicrobial resistance than those isolated from NRM. Furthermore, *E. faecalis* was a predominant species among *Enterococcus* spp. in BMM, showing high virulence characteristics. Taken together, controlling and monitoring *E. faecalis* harboring *esp*, *gelE*, and *cylA* genes could be the best intervention strategy for eliminating bovine mastitis, leading to improved public health.

## Figures and Tables

**Table 1:** Distribution of virulence genes among *Enterococcus faecalis* isolates from bovine mastitis milk (BMM) and bovine normal raw milk (NRM).

Milk Type	No. of Isolates	No of Genes Encoding Virulence Factors (%)			
		*esp* ***	*asa1*	*gelE* ***	*cylA* ***
BMM	70	38 (54.3%)	50 (71.4%)	60 (85.7%)	21 (30.0%)
NRM	20	5 (25.0%)	16 (80.0%)	12 (60.0%)	0

*** indicates significant difference in virulence genes between bovine mastitis milk and normal raw milk (*p* < 0.0001; Pearson’s chi-square test). BMM: bovine mastitis milk, NRM: bovine normal raw milk.

**Table 2:** Correlation between virulence phenotype and genotype of *Enterococcus faecalis* and *Enterococcus faecium* isolated from bovine mastitis milk (BMM) and bovine normal raw milk (NRM).

Virulence Factor	Encoding Gene	Phenotype and Genotype of Isolates from BMM and NRM							
		P+G+		P−G+		P+G−		P−G−	
		BMM	NRM	BMM	NRM	BMM	NRM	BMM	NRM
Strong biofilm formation (=OD > 0.208)	*esp* *	35	5	3	0	18	6	25	30
	*asa1*	37	5	14	11	16	6	14	19
	*gelE* *	43	6	18	6	10	5	10	24
Gelatinase	*gelE* *	18	2	43	10	0	0	20	29
Hemolysin	*cylA*	2	0	19	0	0	0	60	41

* indicates significant associations between virulence factors and related encoding genes (*p* < 0.05; Pearson’s chi-square test). BMM: bovine mastitis milk, NRM: bovine normal raw milk, P+: phenotypically expressed, P−: phenotypically not expressed, G+: encoding gene detected, G−: encoding gene not detected.

**Table 3:** Antimicrobial resistance of *Enterococcus faecalis* and *Enterococcus faecium* isolates from bovine mastitis milk (BMM) and bovine normal raw milk (NRM).

Antimicrobial Agents	No (%) of Antimicrobial Resistance Isolates from BMM and NRM					
	BMM			NRM		
	*E. faecalis* (*n* = 70)	*E. faecium* (*n* = 11)	Total (*n* = 81)	*E. faecalis* (*n* = 20)	*E. faecium* (*n* = 21)	Total (*n* = 41)
AMP	0	0	0	0	0	0
C	16 (22.9%)	1 (9.1%)	17 (21.0%)	0	0	0
CIP	0	1 (9.1%)	1 (1.2%)	0	0	0
DOX	3 (4.3%)	1 (9.1%)	4 (4.9%)	0	0	0
TET	45 (64.3%)	3 (27.3%)	48 (59.3%)	4 (20%)	3 (14.3%)	7 (17.1%)
ERY	19 (27.1%)	3 (27.3%)	22 (27.2%)	1 (5%)	13 (61.9%)	14 (34.1%)
N	0	1 (9.1%)	1 (1.2%)	0	0	0
RIF	11 (15.7%)	4 (36.4%)	15 (18.5%)	1 (5%)	5 (23.8%)	6 (14.6%)
VAN	0	0	0	0	0	0
MDR	12 (17.1%)	1 (9.1%)	13 (16.0%)	0	2 (9.5%)	2 (4.9%)

BMM: bovine mastitis milk, NRM: bovine normal raw milk, AMP: ampicillin, C: chloramphenicol, CIP: ciprofloxacin, DOX: doxycycline, TET: tetracycline, ERY: erythromycin, N: nitrofurantoin, RIF: rifampicin, VAN: vancomycin, MDR: multidrug resistance.

## Data Availability

Not applicable.

## Supplementary Materials

The following supporting information can be downloaded at: https://www.mdpi.com/article/10.3390/ani12111407/s1, Table S1: Polymerase chain reaction primers and product sizes for the detection of *Enterococcus faecalis* and *Enterococcus faecium* virulence genes.
