# Supplementary material for: Prevalence and Virulence Characteristics of Enterococcus faecalis and Enterococcus faecium in Bovine Mastitis Milk Compared to Bovine Normal Raw Milk in South Korea
Source: Animals (Basel). 2022 May 30;12(11):1407. doi: 10.3390/ani12111407 (PMC9179290; doi:10.3390/ani12111407)
Supplement: Supplementary file 1 [file animals-12-01407-s001.zip › animals-1740663-supplementary.pdf]

Supplementary data

**Supplementary Table S1**. Polymerase chain reaction primers and product sizes for the detection of *Enterococcus faecalis* and *Enterococcus faecium* virulence genes.

| Gene | Oligonucleotide Sequence (5′ to 3′) | Product Size (bp) | Annealing Temperature (°C) | Reference |
|---|---|---|---|---|
| *esp* | AGA TTT CAT CTT TGA TTC TTG G<br>AAT TGA TTC TTT AGC ATC TGG | 510 | 52 | [17] |
| *asa1* | GCA CGC TAT TAC GAA CTA TGA<br>TAA GAA AGA ACA TCA CCA CGA | 375 | 56 | |
| *gelE* | TAT GAC AAT GCT TTT TGG GAT<br>AGA TGC ACC CGA AAT AAT ATA | 213 | 56 | |
| *cylA* | ACT CGG GGA TTG ATA GGC<br>GCT GCT AAA GCT GCG CTT | 688 | 56 | |
